# Spasticity Assessment Based on the Maximum Isometrics Voluntary Contraction of Upper Limb Muscles in Post-stroke Hemiplegia

**DOI:** 10.3389/fneur.2019.00465

**Published:** 2019-05-07

**Authors:** Hui Wang, Pingao Huang, Xiangxin Li, Oluwarotimi Williams Samuel, Yun Xiang, Guanglin Li

**Affiliations:** ^1^CAS Key Laboratory of Human-Machine Intelligence-Synergy Systems, Shenzhen Institutes of Advanced Technology, Chinese Academy of Sciences, Shenzhen, China; ^2^Shenzhen College of Advanced Technology, University of Chinese Academy of Sciences, Shenzhen, China; ^3^The Rehabilitation Department, Shenzhen Sixth People's Hospital (Nanshan hospital), Shenzhen, China

**Keywords:** spasticity assessment, post-stroke, maximum isometrics voluntary, voluntary activation, reliability

## Abstract

**Background:** The assessment of muscle properties is an essential prerequisite in the treatment of post-stroke patients with limb spasticity. Most existing spasticity assessment approaches do not consider the muscle activation with voluntary contraction. Including voluntary movements of spastic muscles may provide a new way for the reliable assessment of muscle spasticity.

**Objective:** In this study, we investigated the effectiveness and reliability of maximum isometrics voluntary contraction (MIVC) based method for spasticity assessment in post-stroke hemiplegia.

**Methods:** Fourteen post-stroke hemiplegic patients with arm spasticity were asked to perform two tasks: MIVC and passive isokinetic movements. Three biomechanical signals, torque, position, and time, were recorded from the impaired and non-impaired arms of the patients. Three features, peak torque, keep time of the peak torque, and rise time, were computed from the recorded MIVC signals and used to evaluate the muscle voluntary activation characteristics, respectively. For passive movements, two features, the maximum resistance torque and muscle stiffness, were also obtained to characterize the properties of spastic stretch reflexes. Subsequently, the effectiveness and reliability of the MIVC-based spasticity assessment method were evaluated with spearman correlation analysis and intra class correlation coefficients (ICCs) metrics.

**Results:** The results indicated that the keep time of peak torque and rise time in the impaired arm were higher in comparison to those in the contralateral arm, whereas the peak torque in the impaired side was significantly lower than their contralateral arm. Our results also showed a significant positive correlation (*r* = 0.503, *p* = *0.047*) between the keep time (t_k_) and the passive resistant torque. Furthermore, a significantly positive correlation was observed between the keep time (tk) and the muscle stiffness (*r* = 0.653, *p* = 0.011). Meanwhile, the ICCs for intra-time measurements of MIVC ranged between 0.815 and 0.988 with one outlier.

**Conclusion:** The findings from this study suggested that the proposed MIVC-based approach would be promising for the reliable and accurate assessment of spasticity in post-stroke patients.

## Introduction

Spasticity, commonly defined as motor disorder, can be characterized by velocity-dependent increase in muscle tone with exaggerated tendon jerks that will affect the muscle voluntary activation (1). It has been reported in previous studies that about 40% of post-stroke survivors suffer from spasticity (2, 3), leading to a huge burden on a large number of patients and challenges to the care givers (4, 5). To treat patients with spasticity, a number of different approaches such as local botulinum toxin injection, physical and occupational therapies, electrical neuro stimulation, and surgical interventions, have been commonly used in clinic (2, 6, 7). While the clinical practices have showed that these approaches are effective for spasticity treatments, their clinical efficacy would be further improved if the spasticity assessments are more reliable and accurate. Currently, the commonly used spasticity treatments are the clinical scale methods such as Ashworth Scale and Modified Ashworth Scale (MAS) which could provide some useful information on whether spasticity exists or not and what the severity of spasticity are with several levels (such as 0–4) (8, 9). The most widely applied MAS method is relatively easy to implement, but its assessment outcomes could be only used for passive movements' assessment (10, 11) and greatly depend on the physicians' experience (12, 13). With these issues, the MAS method has been questioned by several researchers over time (13, 14) These discrete level assessment methods could roughly group spasticity, however, they could not provide sufficiently reliable or accurate information for assessing spasticity that would be necessary for guiding spasticity treatments (15, 16).

With the limitations of the clinical scale methods, some methods based on the analysis of features associated with neurophysiologic/biomechanical measurements have been proposed in previous studies for accurately evaluating the spasticity in patients (17–22). It should be noted that most of the previous neurophysiologic/biomechanical spasticity assessment methods will be greatly affected by individual differences among patients and partial side effect (23, 24). In order to overcome these limitations, a number of quantitative methods have been developed for spasticity assessment in patients (25). For examples, the H-reflex, H/M ratios, and dynamic electromyogram (EMG) response to mechanical stimuli based approaches, have been proposed and investigated for spasticity assessment in patients (17, 26). Although these neurophysiological based methods appear to be promising, they are still limited by several factors including inadequate electrode placement, electrode-skin resistance, and physiological status of the muscles amongst others (23). In addition, these neurophysiology-based methods usually lack a direct correlation between the neurophysiological assessment outcome and the clinical scale outcome, which makes the clinicians be difficult to assess the spasticity status of patients (27). Further, biomechanical methods driven by the initiation of different kinds of muscular movements including isokinetic, isometric, isotonic have equally been used for assessment of muscle characteristics especially via rehabilitation training (28). And the maximum isometrics voluntary contraction (MIVC) used in assessing the characteristics of voluntary muscle activation is considered as a useful approach for quantifying the neuromuscular properties of the spastic muscles (29). Although isokinetic test method is considered as a standard approach for assessing the stretch reflex with respect to spasticity only in the context of passive movement, it cannot discriminate between neural component and muscle component (21). Additionally, it is unknown whether the MIVC method could offer a useful measure for assessing the neuromuscular properties of spastic muscles.

Currently, the peak resistive torque and stiffness that are calculated with isokinetic dynamometry are considered as the “gold standard” for the evaluation of spasticity (16) and even the most existing spasticity assessment approaches are based on the evaluation of the neurophysiologic/biomechanical response to stretch reflexes (30). However, the muscle activation characteristics with voluntary contraction are completely neglected in these methods (31). It is unknown whether the voluntary movements of spastic muscles are useful in the reliable assessment of muscle spasticity. After all, the evaluation of the muscles response to voluntary movements (voluntary muscle activation) have rarely been considered to date, and the relationship between the spastic muscle tone and muscle voluntary activation remains unclear (31, 32). In addition, it is also unclear what the relationship between the spastic muscle tone and muscle voluntary activation are.

In this study, a new spasticity assessment method based on MIVC was proposed and its performance in evaluating the spasticity status in post-stroke hemiplegia with an upper limb spasticity was investigated. Current method was compared with the conventional passive stretch method as well as the MAS method. In addition, three biomechanical features (peak reflex torque, keep time of the peak torque, and rise time of the peak torque) derived from MIVC were proposed to quantitatively assess upper limb spasticity. Additionally, further investigations were conducted to evaluate the changes in the voluntary muscle activation properties between the impaired and non-impaired arms, using correlation between the features from current method and conventional passive stretch reflex approach in chronic stroke survivors with spasticity. Criterion validity was examined as convergent construct validity (using the Spearman's correlation coefficient) and concurrent validity (using analysis of variance) (11). The reliability of the MIVC measurements was further evaluated using repeated measurements intra class correlation coefficients (ICCs).

## Materials and Methods

### Participant Information

In this study, we enrolled a total of 14 chronic post-stroke hemiplegia (11 males and 3 females) with different degrees of elbow flexor spasticity (MAS = 1, 1+, 2), age of 47.36 ± 6.54 years, and an average post-stroke time of 6.18 ± 2.47 months. All the participants were observed to be in their post stroke recovery stages (Time since stroke is more than 1 month), and their summarized demographic information was listed in Table 1. The inclusion criteria for subjects were (1) hemiplegia secondary to a single ischemic or hemorrhage stroke; (2) at least 1 months post-stroke; (3) elbow flexor spasticity of the impaired side < 3 (rated by MAS); (4) being able to understand and follow instructions related to the experiment; and (5) being able to give written informed consent. The exclusion criteria were (1) a history of multiple strokes or bilateral involvement; (2) presence of muscle contraction that would limit full elbow range of movement on the impaired side; (3) existence of function failure in important organs such as heart, lung, liver, and kidney. The experiment was approved by the human research ethics committee of the Shenzhen Nanshan hospital and all the subjects gave written informed consent prior to their participation in the study. In addition, all the experiments were performed in accordance with the relevant guidelines and regulations.

**Table 1:** Summarized demographic information of all the subjects according to MAS (*N* = 14)^*^.

**MAS scores**	**No. subjects**	**Impaired side**	**Age(years)**
1	5	4R/1L	49.6 ± 9.7
1+	5	2R/2L	45.3 ± 3.1
2	4	3R/2L	45.5 ± 4.9

* *Age was shown in Mean ± SD, and the MAS as range, impaired side (Right or Left) and gender (Male or Female) as distribution*.

### Experiment Procedure

**Figure 1:**
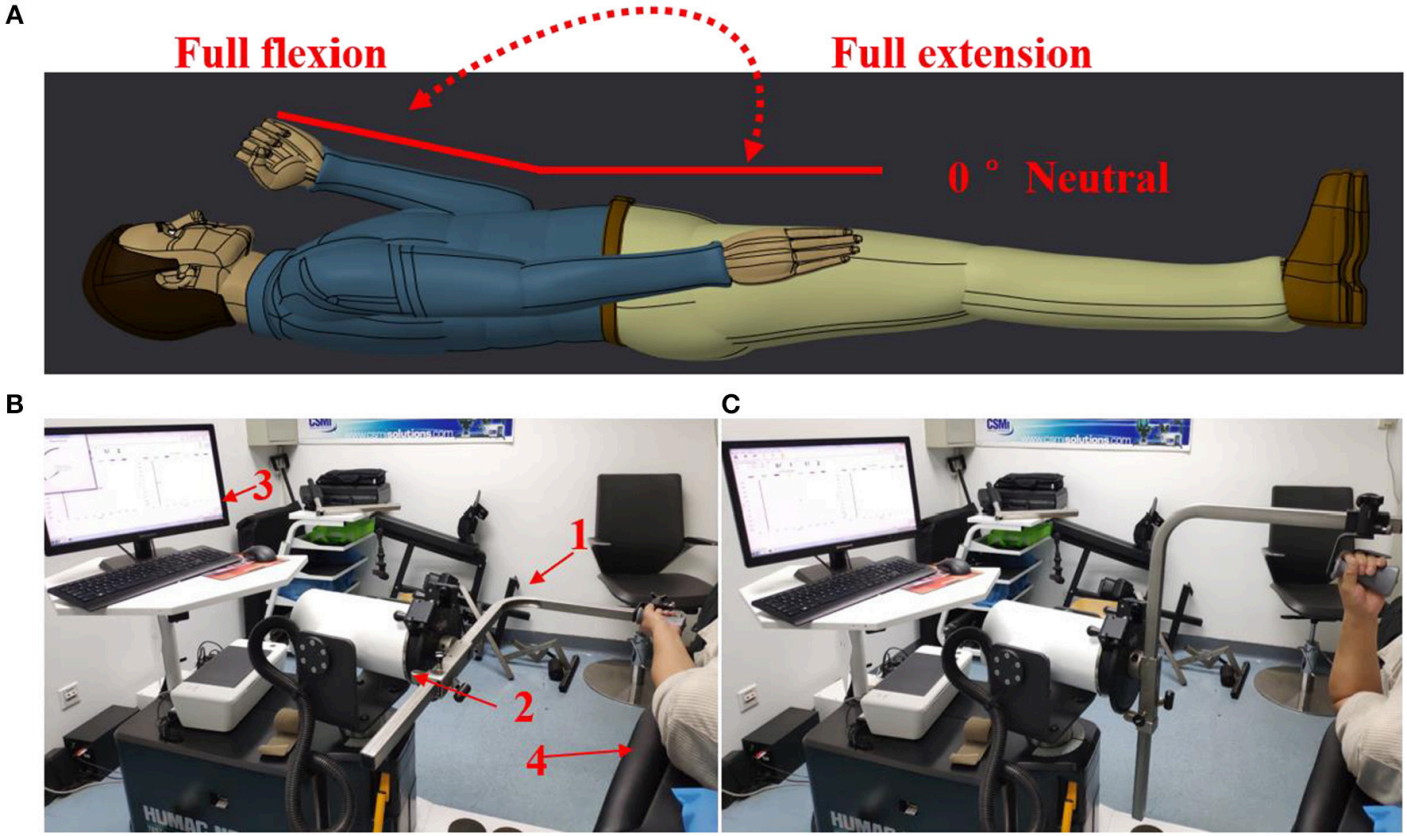
Experimental setting. **(A)** The definition for the range of movement with elbow joint. **(B)** Maximum isometric voluntary contraction at an elbow joint angle of about zero degree Test for both arms and HUMAC NORM device introduction, 1: elbow/shoulder handle, 2: dynamometer, 3: computer display and control platform, 4: examination bed; **(C)** Elbow passive isokinetic contractions test for impaired limb.

A commercial motor function rehabilitation system HUMAC NORM (Computer Sports Medicine Inc. CSMI, USA) was used to record biomechanical signals (speed, torque, and position) in the study. The body weight and other necessary features of each subject were also regularly recorded before the experiments. During an experiment, each subject lay comfortably on an examination bed, and the MIVC signals associated with the impaired and non-impaired arms were recorded by HUMAC NORM device, as shown in Figure 1. Each subject was asked to hold the handle of HUMAC NORM device with a normal force and to perform the maximum isometric voluntary contraction at an elbow joint angle of about zero degree for three sessions. When one session was finished, their arms of a subject were relaxed for a rest at least 15 s before doing next session. In order to minimize the effect of muscle fatigue on the spasticity assessment, the MIVC signal recordings from the third MIVC trial was excluded. Subsequently, the range of the elbow joint angle was tested in a rest session after their arms have been passively stretched to avoid muscle fatigue. Then, they further performed three passive isokinetic contractions using their impaired limb with at least 20 s rest session in between. And three constant passive stretch speeds of elbow flexors, 60°/s, 40°/s, and 20°/s, were considered in the study. For each stretch speed, the participants repeated three trials of passive isokinetic contraction. The onset elbow angle was about zero degree and the end position was the approximate maximum elbow movement angle.

### Neuromechanical Parameters (*T*_*p*_, *T*_*k*_, *T*_*r*_)

**Figure 2:**
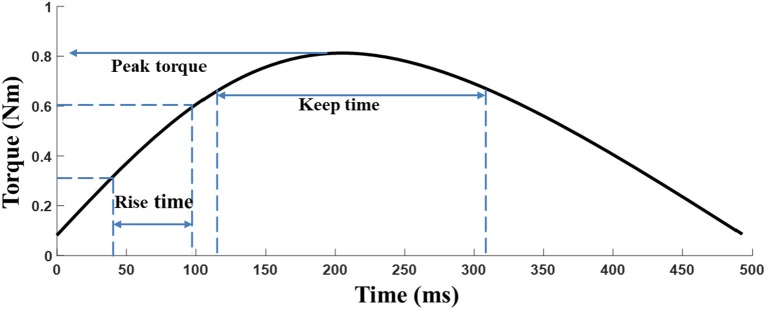
Description of the neuromechanical parameters for the MIVC.

The elbow torque acquired with the HUMAC NORM device from the MIVC task was filtered by a 3rd order Butterworth low pass filter with a cutoff frequency of 1 Hz. The peak torque (*T*_*p*_) was defined as the maximum torque of an isometric maximum voluntary contraction of the elbow flexors. *T*_*p*_ represents the muscle strength of the participants and the keep time (*T*_*k*_) was defined as the duration for which the muscle strength was maintained (above 80%^*^maximum torque), and *T*_*k*_ equally indicates the muscle endurance (25). The rise time (*T*_*r*_) is defined as 0.1^*^ maximum torque to 0.8^*^ maximum torque for a given trial, where *T*_*r*_indicate muscle power (Figure 2). The peak torque was normalized by individual body weight to reduce the subject individual differences among patients (26).

**Figure 3:**
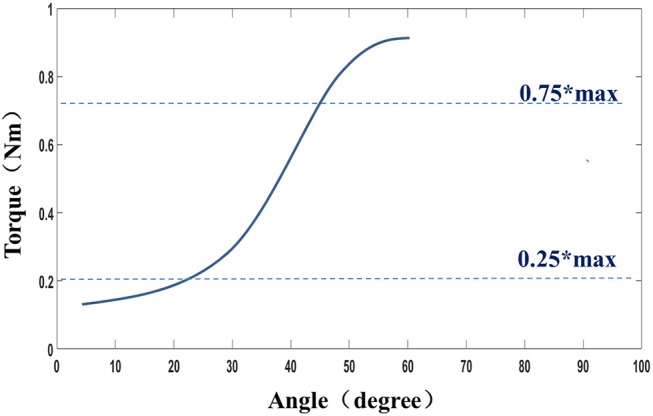
Definition of the stiffness.

The muscle stiffness characteristic was determined by fitting a slope to the stress–stretch data by means of linear regression between the points of 0.25^*^maximum stress and 0.75^*^maximum stress for a given trial (Figure 3). This portion of the typically sigmoidal response was well-described by the linear regression, as verified in the results section (27).

### Statistics

The signal processing task was performed with MATLAB R2015b (Math Works) programming tool and all statistical analyses were carried out with SPSS (version 17.0) software. Meanwhile, the paired sample *T*-test was applied to examine if there was a significant difference between impaired side and non-impaired side. Criterion validity was investigated as convergent construct validity (using Spearman's correlation Coefficient) and concurrent validity (using analysis of variance and Tukey's *post-hoc* test). Correlations between the proposed features of MIVC (*T*_*p*_,*T*_*k*_,*T*_*r*_), MAS and biomechanical measures (peak reflex torque and reflex stiffness) were analyzed using Spearman's coefficient. Statistical significance level was set at *p-value* < 0.05 and false discovery rate (FDR) analyses were provided with FDR correction (*p*-value was convert to *q*-value, and *q* = p^*^n/*rank*, in the equation n denotes the comparison time and rank denotes the order of *p*-value from small to big). The reliability of MIVC measurements was evaluated using repeated measurements ICCs with 95% confidence intervals (CIs). The ICC was calculated using a two-way mixed-effect model with an agreement coefficient. ICC values would vary from 0 to 1.

## Results

For each subject, the properties of maximum isometric voluntary contraction from the impaired and non-impaired sides were analyzed, and the MIVC Features (*T*_*p*_,*T*_*k*_,*T*_*r*_) were compared between the impaired and non-impaired sides. Then the effect of the velocity on the passive stretch associated with the respective muscles was investigated. Further, the relationship that exists between the proposed features of MIVC, MAS, and biomechanical measures was examined using correlation and linear regression analysis techniques. Finally, the reliability of the MIVC measurements was assessed with ICCs and Bland-Altman plot.

### The Properties of MIVC on the Impaired and Non-impaired Sides

**Figure 4:**
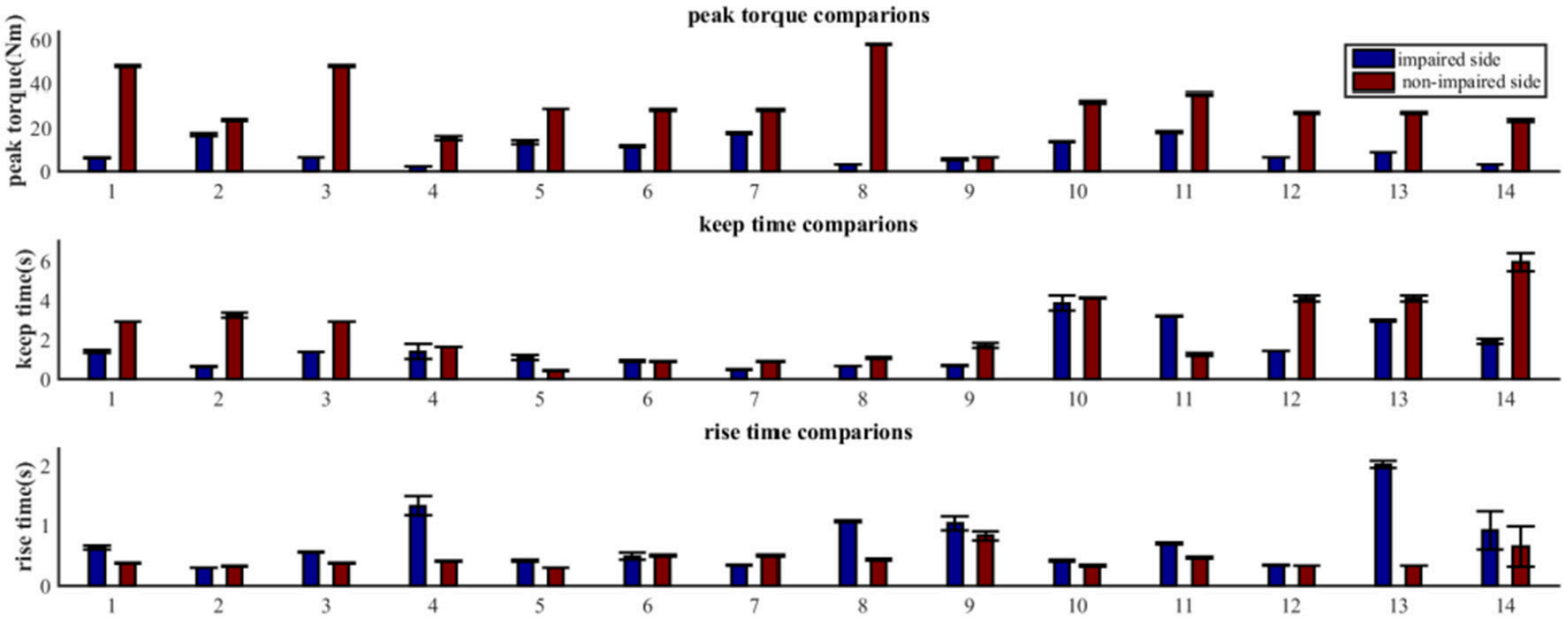
Comparative analysis of MIVC features between the impaired and non-impaired arms across all the 14 recruited subjects (Mean ± SEM).

As shown in Figure 4, the MIVC features (peak torque, keep time, rise time) were significantly different between the impaired and non-impaired arms for each subject. Generally speaking, for all the subjects, the mean of the peak torque *T*_*p*_ on their impaired side was less than that on their non-impaired side. For 11 of 14 subjects, the keep time of peak torque *T*_*k*_ on their impaired side was lower in comparison to that on their non-impaired side. And for 12 of 14 subjects, compared to their non-impaired side, the rise times of peak torque *T*_*r*_ were greater on their impaired side.

Our statistical analysis results show that the difference between the impaired and non-impaired side (Table 2) with respect to the three MIVC features was significant. During the maximum isometric voluntary contraction, the mean of the peak torque *T*_*p*_ on the impaired side (0.147 ± 0.086 Nm) was less than that on the non-impaired side (0.465 ± 0.202 Nm) (*p* < 0.001, *q* < 0.001). Meanwhile, the mean of the keep time of peak torque *T*_*k*_ was lower on the impaired side (0.147 ± 0.086 s) than on the non-impaired side (0.465 ± 0.202 s) (*p* = 0.037, *q* = 0.037). Furthermore, the mean of the rise time of peak torque *T*_*r*_ was greater on the impaired side (0.147 ± 0.086 s) than on the non-impaired side (0.465 ± 0.202 s) (*p* = 0.029, *q* = 0.044). The *p*-values were converted to *q* value with FDR correction, and the significant test results remained the same.

**Table 2:** Comparison of MIVC features between impaired side and non-impaired side.

**Features**	**Impaired side (Mean ± SEM)**	**Non-impaired (Mean ± SEM)**	**Impaired-Non-impaired (*P*-value)**	**FDR corrected (*q*-value)**
*T*_*p*_	9.614 ± 1.492	30.660 ± 3.566	< 0.001	< 0.001
*T*_*k*_	1.586 ± 0.281	2.524 ± 0.438	0.037	0.037
*T*_*r*_	0.757 ± 0.129	0.441 ± 0.039	0.029	0.044

### Analysis Based on Velocity-Dependent Responses of Passive Stretch

**Figure 5:**
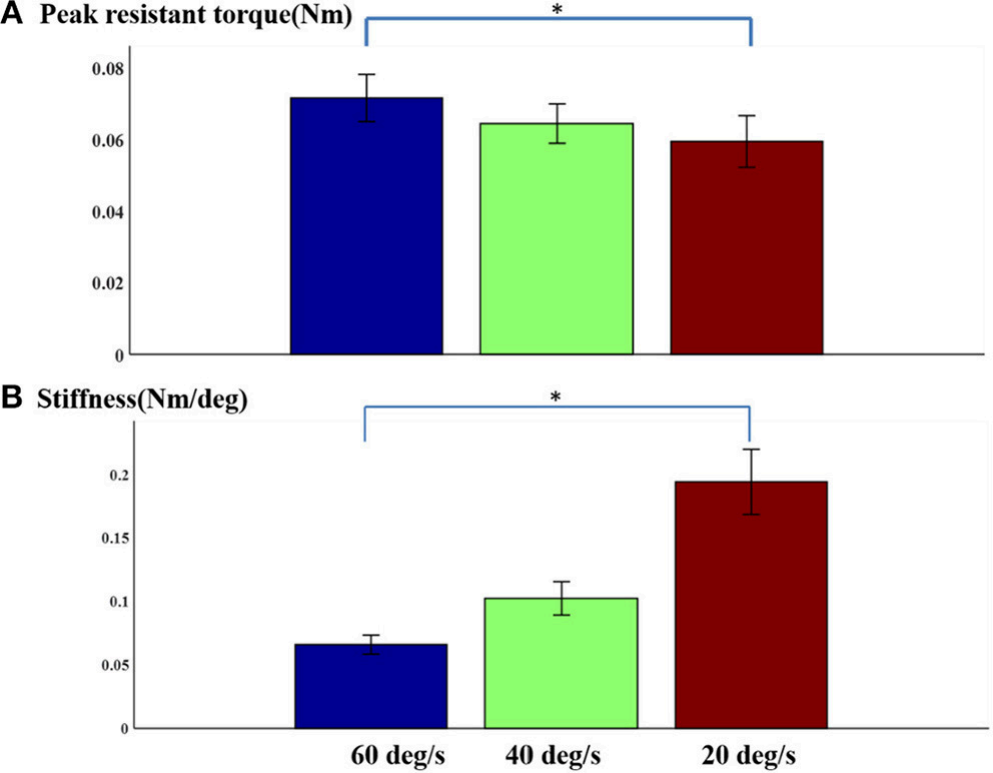
**(A)** Normalized peak torque and **(B)** stiffness at three speeds Mean ± SEM, * represents statistical significance).

In general, there was no obvious change in velocity-dependent mechanical response of passive stretch with peak torque. In fact, the results obtained from a repeated one-way ANOVA analysis did not reflect a meaningful effect of stretch velocity for peak torque response [*F*_3, 4_ = 0.89, *p* = 0.42] (Figure 5A). It was observed that the response at higher velocities showed greater individual variation as indicated by the SEM bars. The slight differences in passive resistive torque between the three stretch velocities indicated that the stretch reflex of the muscle may be induced by the all the three stretch velocities. And the elbow angular velocity threshold for inducing stretch reflex response was lower than 20°/s. Additionally, it can be observed from Figure 5A that the velocity-dependent mechanical response in passive stretch increases correspondingly with the stiffness. And the outcome of one-way repeated ANOVA indicated that there was a significant effect in stretch velocity for stiffness response. Figure 5B shows the direct relationship between the stiffness in elbow flexors and the velocity. Similarly, there was a significant effect of stretch velocity [*F*_3, 4_ = 14.7, *p* < 0.001]. The response at higher velocities represented greater individual variation as shown by the SEM bars.

### Correlation Between MIVC-Features and Biomechanical Assessments

**Figure 6:**
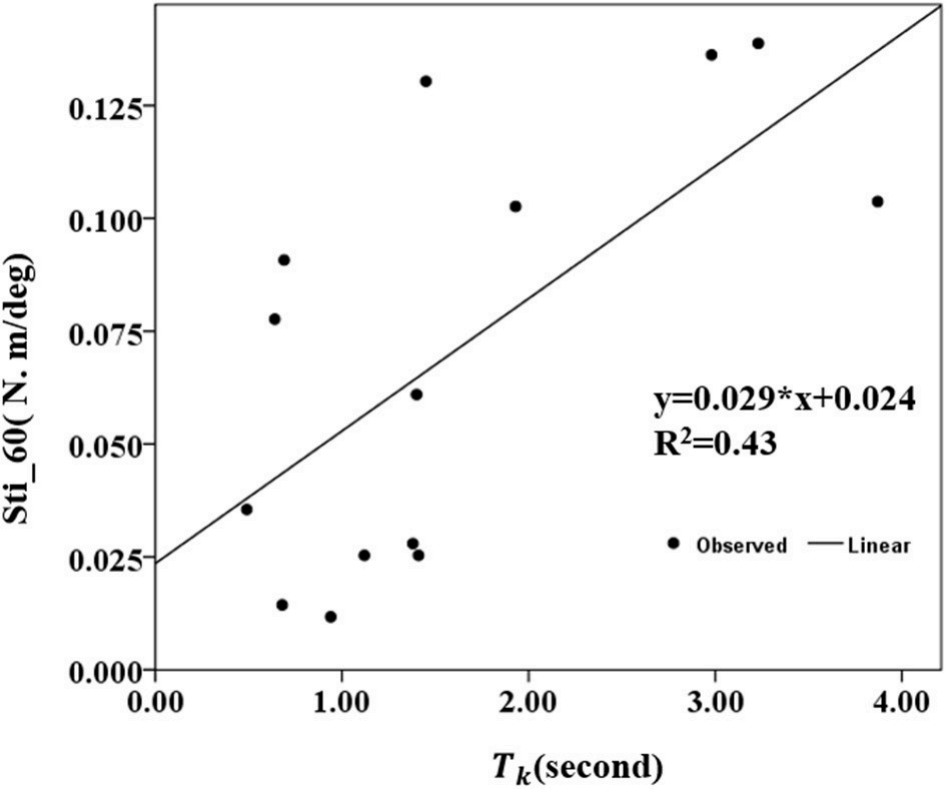
Conelation between the stiffness of 60°/s and the keep time *(T*_*k*_*)* of the MIVC with linear regression analysis.

The correlation coefficients between the MIVC-features and stretch measurements were computed and presented in Table 3. It can be seen from Table 3 that there was a strong positive relationship between the peak resistant torque from the passive stretch test at 60°/s and the *T*_*k*_ from the MIVC (*r* = 0.503, *p* = 0.047). In addition, a strong positive relationship between the muscle stiffness from the passive stretch velocity at 60°/s and the *T*_*k*_from the MIVC (*r* = 0.653, *p* = 0.011) was also obtained as shown in Figure 6. No other association between the MIVC features and passive stretch measurements was observed. It should be noted that no significant correlation between MIVC indexes and the MAS was observed while only the correlation between the *T*_*p*_ and the MAS was approximate significant (*r* = −0.503, *p* = 0.061). Further, evaluation of the associations between the MAS scores and the passive stretch measurements did not confirm any correlations results obtained via Spearman coefficients (*P*).

**Table 3:** Spearman correlation analysis among MIVC features, stretch reflex features, and MAS (Correlation coefficient and *P*-value).

**Variables**	**MAS**	**tp_60**	**sti_60**	**tp_40**	**sti_40**	**tp_20**	**sti_20**
*T*_*p*_	−0.503 (0.067)	0.209 (0.474)	0.270 (0.350)	0.257 (0.375)	0.165 0.573)	0.257 (0.375)	0.196 0.503)
*T*_*k*_	0.084 (0.776)	0.503^*^ (0.047)	0.653^*^ (0.011)	0.424 (0.131)	−0.147 (0.615)	0.516 (0.059)	0.196 (0.503)
*T*_*r*_	0.000 (1)	−0.152 (0.604)	0.037 (0.899)	−0.148 (0.615)	−0.099 (0.736)	−0.183 (0.532)	0.258 (0.374)

* *tp_60, tp_40, and tp_20 individually represent the peak torque from passive stretch of three velocity 60°/s, 40°/s, 20°/s. sti_60,sti_40 and sti_20 are similar*.

### Reliability of MIVC Measurements With Intra Class Correlation Coefficient

Generally, our experimental results showed that the reliability of the MIVC measures was very fine. The ICC results were presented in Table 4. It can be seen from Table 4 that the ICC values ranged between 0.653 and 0.990, and the Peak torque of the MIVC showed the best reliability with a coefficient of 0.99 (excellent reliability) for the non-impaired limb, and 0.96 for the impaired limb. The keep time of the MIVC also showed great reliability characteristics with a coefficient of 0.82 for the impaired limb and 0.98 for the non-impaired limb. The rise time of the MIVC showed the worst reliability with a coefficient of 0.65 for the non-impaired arm.

**Table 4:** The repeated measurements intra class correlation coefficients (ICCs) with 95% confidence intervals (CIs) of the MIVC features.

Feature^*^	**First measurement (mean ± SEM)**	**Second measurement (mean ± SEM)**	**ICC (95% CI)**
*a*_*T*_*p*_	9.202 ± 1.438	8.471 ± 1.321	0.962 (0.881–0.988)
*h*_*T*_*p*_	30.443 ± 3.578	28.709 ± 3.632	0.988 (0.988–0.999)
*a*_*T*_*k*_	1.652 ± 0.30	1.575 ± 0.263	0.815 (0.425–0.941)
*h*_*T*_*k*_	2.271 ± 0.373	2.171 ± 0.344	0.975 (0.922–0.992)
*a*_*T*_*r*_	0.885 ± 0.175	0.639 ± 0.125	0.893 (0.666–0.966)
*h*_*T*_*r*_	0.440 ± 0.039	0.394 ± 0.014	0.653 (−0.081 to 0.889)

* *a_Tp indicates the peak torque from the impaired side, h_Tp indicates the peak torque from the non-impaired side, others are similar*.

**Figure 7:**
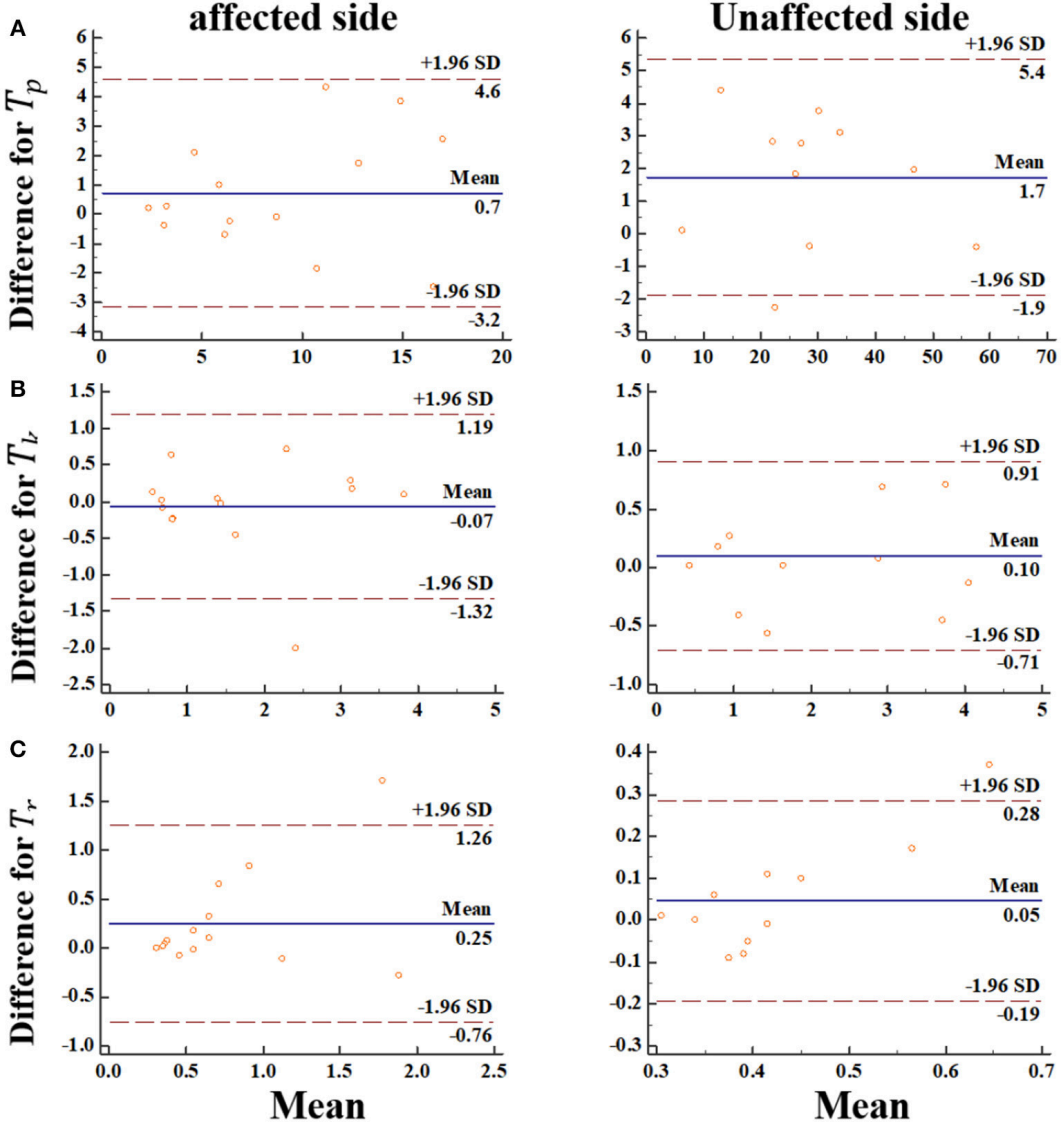
The Bland-Altman plot for the MIVC features, longitudinal axes indicates the mean of two measurements, transverse axes indicates the difference of the two measurements.**(A)** Peak torque *T*_*P*_*;* **(B)** Keep time *T*_*k*_*;* **(C)** rise time *T*_*r*_.

We went further and visualized bias systematically using Bland-Altman graph. In this regard, the Bland-Altman plots indicated that there was no bias for the repeated two measurements (Figure 7). Meanwhile, the data points were distributed equally above and below the zero lines, which indicated no bias. For *T*_*k*_, *T*_*r*_ from the impaired side, only one points was out of the boundary lines (−1.96^*^SD, 1.96^*^SD). For *T*_*p*_, *T*_*r*_ from the non-impaired side, only one points was out of the boundary lines. These results suggested the reproducibility of the MIVC features.

## Discussion

Effective spasticity management usually results to restoration of biomechanics, improvement of motor control, strengthening of weak muscles, and improvement of muscle endurance. Recent evidences suggest that voluntary activation change of the spastic muscle may contribute more to disability than abnormal stretch reflex in post-stroke patient (33). However, only a fraction of the existing related works have focused on muscle voluntary activation with maximum voluntary contraction, thus a study on the relationship between muscle tone and voluntary activation is desirable. To the best of our knowledge, this study might be the first study to systematically investigate and assess spasticity with MIVC features. Our experimental results indicated that the proposed MIVC features would correlate with muscle tone, which were important indicators for spasticity rehabilitation. And the MIVC features were reliable in terms of providing consistent test results. In addition, the relationship between the MIVC and passive stretch movement as well as the MIVC feature differences between the impaired and non-impaired arms were investigated in the study. We found that the biomechanical tests results provided experimental evidence that *T*_*k*_ could be effectively used to assess post-stroke spasticity.

### Relationship Between Passive Stretch Mechanical Features and MAS

A relatively weak relationship was observed between the passive torque, muscle stiffness, and the MAS. This observation is in line with those reported in a number of previous studies (14, 34, 35), which indicates that the MAS based methods might be not a very suitable means for reliably assessing spasticity in patients. A velocity dependent increase in passive resistant torque was equally observed with a peak resistant torque that kept increasing especially at higher passive velocity. For instance, the peak resistant torque from high velocity of 40°/s and 60°/s were found to be larger than the peak resistant torque from low velocity of 20°/s and 40°/s, correspondingly. Interestingly, other previous investigators have reported a progressive increase in biceps brachii resistive torque at stretch velocities >40°/s in normal subjects and patients with spinal cord injury which corroborates the findings from the current study (34). It should be noted that the stretch reflex-mediated response and non-reflex response were not distinguished. Meanwhile, the muscle stiffness increased linearly in response to increasing passive velocity, whereas velocity dependent response was observed. The stiffness strongly correlates with passive resistant at the three levels of passive stretch velocity. These results were also consistent with those reported in some previous studies (36, 37).

### Correlation Between the MIVC Features and Passive Stretch Measurement

Investigations on the correlation between MIVC and passive stretch revealed a fairly strong relationship between the passive torque, stiffness, and the *T*_*k*_, indicating that the proposed method is clinically relevant. The weak relationship that was observed between the passive torque, stiffness, and *T*_*p*_, *T*_*r*_ shows that there is a low association between the measured passive stretch and the muscle strength in the spastic arm. In other words, *T*_*p*_ and *T*_*r*_ may be not suitable for muscle spasticity assessment. At 20°/s, the Peak torque would be low and insensitive to reflex–mediated response, thus accounting for the reflex response and non-reflex response. Hence, this would be a reasonable explanation for the significant correlation observed between MIVC features and passive stretch response at 60°/s, and insignificant correlation between the MIVC features and passive stretch response at 20°/s.

### Voluntary Muscle Activation Between the Impaired Side and Non-impaired Side

With the investigation of the characteristics of the muscles on the impaired arm and that of the contralateral side based on extracted MIVC features (*T*_*p*_,*T*_*k*_,*T*_*r*_), it was interesting in that there was a significant difference between the muscle activation patterns/properties of both arms. In fact, the Peak torque value associated with the impaired arm was found to be significantly smaller compared to that of the non-impaired side (26). Meanwhile, the Keep time was also observed to be significantly smaller on the impaired side than non-impaired side. Additionally, the Rise time was significantly higher on the impaired side than the non-impaired side which is consistent with the results reported in some previous studies (33, 38). In other words, the above discussed results indicated that the muscle strength of the impaired side and the endurance of the impaired side were both reduced.

### Reliability of MIVC Measurements

By examining the reliability of the MIVC features with repeated measurements for spasticity assessment, we found that the relatively high reliability could be achieved with an interclass correlation coefficient of 0.653–0.988. Also, the reliability analysis based on the Bland-Altman plots indicated that the MIVC method is reliable. Although the results were limited to the elbow flexor muscle group, we believe them to be positive enough to use MIVC characters for grading spasticity. If patients are tested with a greater latency between measurements, ratings of spasticity might differ more than in this study. Such differences, however, might be a manifestation of variations in muscle spasticity.

## Limitations

Despite the good performances of the MIVC based features for spasticity assessment, the proposed MIVC method also has some limitations. Firstly, it should be noted that certain post-stroke patients especially those in the soft palsy phase could hardly perform MIVC with their impaired arm because their muscle force would be usually too low to perform any active movement (38). In this regard, the currently investigated MIVC features may not provide optimal results when used to assess the spasticity status of their impaired arm. Secondly, most hospitals often are equipped with dynamometer, but the technical support that is needed to record the time-torque response for MIVC and analyze the data, may be unavailable. Thirdly, as lack of sufficient clinical data and control study, it would be hard to propose the diagnostic criteria for spasticity assessment with MIVC.

## Conclusion

This study provides some experimental evidence that the muscle voluntary activation characterized by Keep time of the Peak torque from the MIVC correlates with severity of spasticity in chronic stroke survivors. The performance of the proposed MIVC method for spasticity assessment was extensively investigated with results revealing its reliability and accuracy based on dataset from14 post-stroke survivors. The findings of this study could provide potential insight on the development of smart intelligent devices that would facilitate efficient spasticity assessment in stroke survivors, which is necessary for active rehabilitation.

## Data Availability

All datasets generated for this study are included in the manuscript and/or the supplementary files.

## Ethics Statement

This study was carried out in accordance with the recommendations of the human research ethics committee of the Shenzhen Nanshan hospital with written informed consent from all subjects. All subjects gave written informed consent in accordance with the Declaration of Helsinki. The protocol was approved by the human research ethics committee of the Shenzhen Nanshan hospital.

## Author Contributions

HW analyzed the data and drafted the manuscript. HW, PH, and YX acquired the data. PH, GL, XL, and OS discussed the idea and experiments of this study and the revised the manuscript.

### Conflict of Interest Statement

The authors declare that the research was conducted in the absence of any commercial or financial relationships that could be construed as a potential conflict of interest.
